# Factors supporting retention of health and wellbeing staff in Aboriginal health services: a strength-based case study

**DOI:** 10.1186/s12960-021-00557-4

**Published:** 2021-03-18

**Authors:** Sara Deroy, Heike Schütze

**Affiliations:** 1 South Coast Women’s Health and Welfare Aboriginal Corporation (Waminda), 122 Kinghorne Street, Nowra, NSW 2541 Australia; 2 School of Public Health and Community Medicine, UNSW Sydney, Sydney, NSW 2052 Australia

**Keywords:** Staff turnover, Staff burnout, Indigenous staff, Aboriginal health worker, Aboriginal community controlled health organisation, Aboriginal medical service

## Abstract

**Background:**

Aboriginal Community Controlled Health Services are fundamental to improving the health and welfare of Aboriginal peoples. A key element that contributes to the effectiveness of these services are Aboriginal health and wellbeing staff. However, Aboriginal health and wellbeing staff often suffer high rates of stress and burnout. Current literature focuses on proposed strategies to increase staff retention in Aboriginal Health Services, yet, there is limited information available showcasing what has actually worked.

**Method:**

This was an intrinsic strengths-based case study of one regional Aboriginal Community Controlled Health Service. Semi-structured research yarning interviews were conducted with past and present staff employed in health and wellbeing roles to highlight the factors that staff felt contributed to their retention.

**Results:**

Ten interviews were conducted between February and April 2018. Six key themes emerged: social accountability, teamwork and collaboration, cultural safety, supervision, professional advancement, and recognition. We add to the literature by identifying the importance of bi-directional communication, and showing that social accountability, teamwork and collaboration, cultural safety, supervision, professional advancement, and recognition continue to be important factors that contribute to health and wellbeing staff retention in Aboriginal Health Services.

**Conclusion:**

This exemplar Aboriginal Health Service may provide insights into future strategies to improve staff retention in other health services.

## Background

This research acknowledges the diversity of Aboriginal and Torres Strait Islander populations in Australia. Throughout the paper, the term ‘Aboriginal peoples’ will respectfully be used to refer to Aboriginal and Torres Strait Islander Australians.

The term ‘Health and Wellbeing Staff’ will be used to refer to a range of positions that support the health and wellbeing of clients such as Aboriginal Health Workers, midwives, social workers, counsellors, caseworkers and community support roles.

Aboriginal and Torres Strait Islander Australians are the longest living culture in the world with a history spanning at least 65,000 years [1]. They have a holistic approach to health and wellbeing, which includes physical and mental health of the individual and the entire community [2]. However, European settlement and colonisation of Australia has resulted in significant disparities in health and wellbeing between Aboriginal peoples compared to other Australians: Aboriginal males have a 10.6 year lower life expectancy; females 9.5 years [3]. Aboriginal people are less likely to access health care when they need it for a range of reasons including feeling unwelcome or afraid, a sense of alienation, fear of discrimination, mistrust in mainstream services, language barriers, and a lack of cultural safety [4].

The Australian Health Practitioner Regulation Agency [5] (page 1) define cultural safety as, “[the] individual and institutional knowledge, skills, attitudes and competencies needed to deliver optimal health care for Aboriginal and Torres Strait Islander Peoples”. To work towards cultural safety, interactions between the health service, staff and community members need to reflect respectful, open and honest relationships, free from bias, racism and assumptions [6]. To address the need for cultural safety in health care and a right to self-determination, Aboriginal Community Controlled Health Services (ACCHS) were established in Australia [2]. Governed and operated by Aboriginal people, for Aboriginal people, ACCHS’s take a holistic approach to health care and offer a range of comprehensive primary health care services for Aboriginal patients [7] who will travel great distances to access culturally safe health services [8].

Aboriginal Health Workers perform a significant role in ACCHS, helping to overcome some of the barriers found in mainstream health services [9]. Aboriginal Health Workers perform clinical, health promotional, educational and leadership roles, and provide a culturally safe and comprehensive approach to primary health care [9]. However, these staff often experience high rates of stress and burnout in their roles [10] due to the extensive demands and expectations both the workforce and community place on them [11]. Pressure from the workplace results from performing a significant role that is not easily interchangeable with other staff [12]; those from the community result from high expectations of the individual to perform their role outside of work hours for friends and family [11]. In addition, high levels of trauma, grief and loss are seen in both the workplace and in community [11]. Inadequate pathways or methods to deal with this stress and pressure can affect health staff in both the short and long term [12].

A literature review on recruitment and retention of health workers in rural areas found that there was a gap in the literature about what factors support the retention of health workers other than general practitioners [13]. Overall the literature tends to focus on proposed strategies to increase staff retention in Aboriginal Health Services; however, there is limited information available showing what has actually worked [14, 15].

Waminda South Coast Women’s Health and Welfare Aboriginal Corporation (Waminda) is an ACCHS on the South Coast of New South Wales, Australia. Between 2015 and 2018 Waminda’s staff retention rate averaged 87%, while the number of staff employed increased from 66 to 110 [16, 17]. Waminda provides services for Aboriginal women, women with Aboriginal children, and women with an Aboriginal partner. Employees are primarily Aboriginal (72%) and non-Aboriginal (28%) females (with the exception of a small male maintenance team) [17]. The aim of this research was to identify the factors supporting the high retention rates of health and wellbeing staff at Waminda.

## Methods

### Study design

This research was a strengths-based qualitative study using a phenomenological intrinsic case study design [18], which explores a specific and unique phenomena/situation, in this case the workplace environment, bound by time, place and context [19]. This allowed for a detailed investigation of a particular case within a social constructionist paradigm [19]. Social constructionism epistemology states that there is not one objective reality of the world, differing realities and meanings are shaped by individual experience [19]. A qualitative design can be used to explore how people understand and interpret their environments and is, therefore, appropriate when studying people’s perceptions of their workplace [20]. A strengths-based approach builds on individual’s skills, strengths, capacity, and treats them as experts of their own lives, resulting in a more tailored and successful outcome [21].

### Participant recruitment

Purposive sampling was used to recruit current or past Waminda Health and Wellbeing staff. Participants were eligible if they were currently employed or had been employed within the past 5 years at Waminda, in a health and wellbeing role with regular client contact (including clinical staff, counsellors, case workers, program workers and managers). An Expression of Interest was distributed to 61 eligible staff by placing a printed copy in each staff members pigeonhole. Three past staff who fit the eligibility criteria were contacted by the Human Resources department and invited to participate in the study. A total of ten participants were recruited for interviews.

### Data collection

‘Yarning’ is a term used by Aboriginal people to mean a talk or conversation, where stories are shared, and knowledge is developed [22]. Research topic yarning is a more formal conversation, focused around a topic [22]. The researcher introduces and concludes the yarn, often with a social yarn (casual catch-up) beforehand [22]. Research topic yarning allows the conversation to always be directed back to the topic of focus. Using yarning as an Aboriginal research method ensures cultural safety by prioritising Aboriginal ways of communicating [22].

SD, a non-Aboriginal researcher who works in an ACCHS, conducted the face-to-face semi-structured research yarning interviews in a private room at Waminda, at a time preferred by the participant, between February and April 2018. Interviews consisted of seven or eight (depending if the participant was a current or past staff member) broad questions exploring the reasons behind why employees remained in their roles at Waminda for the period they were employed (see Box 1 for an outline of the questions). All interviews were audio recorded with consent. Methods were not modified during the study.

Box 1. Yarning interview guide **Current employee**

- When did you begin working at Waminda and what is your role?
- What were your reasons for starting at Waminda?
- What is it that you like about working at Waminda?
- What is it that you do not like about working at Waminda?
- Do you see yourself in this position short-term or long-term?
- What could Waminda do to improve the length of time you intend to stay for?
- What factors about working at Waminda would influence you to leave?
- What else would you like to add?

**Past employee**

- When did you work at Waminda and what was your role?
- What were your reasons for starting at Waminda?
- What was it that you liked about working at Waminda?
- What was it that you disliked about working at Waminda?
- What factors about working at Waminda influenced you to leave?
- What could Waminda have done to improve the length of time you stayed for?
- What else would you like to add?

### Analysis

Interviews were transcribed verbatim and thematic analysis was performed using NVivo version 11 software [23], a program that assists with coding and organising data. Emergent themes were identified using Braun and Clarke’s thematic analysis framework [24], complimented by Creswell’s Data Analysis Spiral [20]. This involved becoming immersed in the data, generating initial codes, identifying overarching themes, reviewing themes, distinguishing themes, and producing a report [24]. SD coded the first few transcripts and created the initial code frame based on repeated themes identified in the transcripts [24]. HS then reviewed the framework and dominant themes to identify differing or additional insights or meanings, which informed the subsequent analysis.

A number of steps were taken to increase the rigour of the study. SD set aside extra time prior to the commencement of the research yarning interviews to ensure participants felt comfortable and to build rapport with participants [25]. To ensure credibility of the data analysis participants were provided a summary sheet of the coding on their interview so they could verify if the interpretations were correct. This process known as member checking or participant validation, is considered one of the most important steps to ensure credibility [25]. This method was considered appropriate considering all participants were literate. Researcher researcher triangulation was also employed [25]. Both authors used a social constructionist lens for analysis ensuring the themes reflected participant views. Discrepancies were settled through discussion and consensus to reduce researcher bias. Additionally, an Aboriginal academic reviewed the themes to ensure they reflected Aboriginal ways of thinking. Data saturation, the point when no new information was discovered assuring that further data collection and analysis would only yield similar findings, was reached [25].

## Results

Ten research yarning interviews were conducted between February and April 2018. Interviews lasted between 12 and 50 min (average 23 min).

### Participants

Participants included nine current employees (including three who had left and returned to work at the service) and one past employee of Waminda. Seven participants identified as Aboriginal. All participants were female, six were aged 35–44 years. Participants included three clinical staff, two counsellors, one caseworker, three community support staff and four members of management (note some staff performed multiple roles). A summary of participant role information is displayed in Table 1. Participant information has been aggregated for this table to ensure participants remain unidentifiable.

**Table 1:** Participant information

Description	*N*
Aboriginal and/or Torres Strait Islander employees	7
Current employees (including three employees who left and returned)	9
Past employees	1
Clinical role	3
Counsellor role	2
Caseworker role	1
Community support role	3
Management role	4
Median age of participants	35–44 years

Six central themes emerged and are discussed further below: social accountability, teamwork and collaboration, cultural safety, supervision, professional advancement and recognition. Specific activities identified to support staff retention at Waminda are outlined in Table 2.

**Table 2:** Summary table of staff retention strategies identified at Waminda

Theme	Identified staff retention strategies/actions/activities at Waminda
Social Accountability	Ensuring the work is guided by community needs
	Bi-directional, open communication between decision makers, staff and community members
Teamwork and collaboration	Cross training; having staff work in different roles and programs
	Encouraging collaboration with external services
Cultural safety	Embedding Aboriginal cultural ways of doing
	Support staff as both a staff member and community member
	Guidance from Cultural mentors, Elders and Cultural Committee
	Leave for cultural events or in-house celebrations
	Safe environment to identify and practice culture
Supervision	Managers/supervisors regularly following up on individual staff wellbeing
	Regular formal operational supervision
	Regular clinical supervision
	Strong leadership and support from the CEO
Professional advancement	Regular opportunities to study or attend workshops and conferences
	Ability to study during work hours
	Recognition of Aboriginal cultural knowledge
	Succession planning for Aboriginal staff
Recognition	Encouraging staff and providing access to programs within the organisation
	Providing team bonding and self-care activities for all staff
	Retaining the dignity, trust and respect of staff who lost jobs due to funding cuts; offering staff their positions back when funding returned

### Social accountability

Staff valued that Waminda operated in a way that was accountable to the community, listening to what the community needed and responding to those needs. Staff saw their roles as long-term commitments, where they could make meaningful connections and give back to the community. One respondent highlighted how she left the organisation she was working at, to join Waminda because of Waminda’s focus on the community.

*“…the management…are very innovative and creative in what they do and responding to community need …that’s what community control is all about, it’s about responding to community need. …I did a lot of community consultation so what I noticed is [I] go out to community and consult with the women and bring it back in to the service and basically the leadership and management would take that on board and, where possible, try and manoeuvre programs or create programs from that. So, in terms of responding to community need and self-determination and having a community focus—that’s what really drew me to the service…”* (HWW06).

**Fig. 1:**
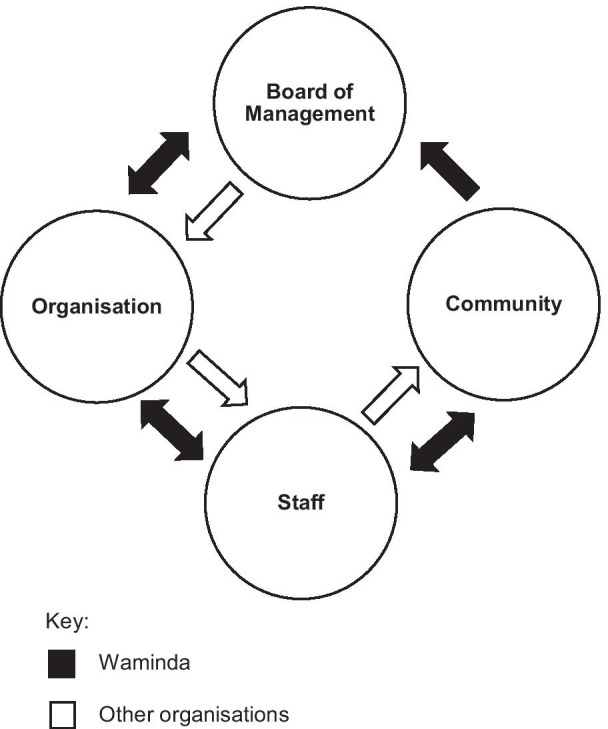
Schematic representation of Waminda’s bi-directional relationships compared to uni-directional relationships possibly seen in other organisations

This bi-directionality in communication and relationships between the Board of Management, the Chief Executive Officer (CEO), staff members, and the community are illustrated in Fig. 1. This figure demonstrates the interconnected relationships at Waminda which differentiates Waminda to other organisations with linear relationships, where the community would receive services from an organisation without their input and consultation.

Four respondents had left their positions at Waminda, with three later returning to the organisation. One staff member left for family reasons, but kept regular contact with Waminda’s CEO and returned to the organisation as soon as they could. *“I rang [name] and I told [name] … I wanna move back to Nowra because … Waminda makes me feel like I am … more connected to this community.” “I feel like I want to give back to my Community and this is the way I can”* (HWW01).

One respondent described finishing their studies, while at Waminda, but there was no position free for that role at Waminda. She applied for the position as soon as it became available: *“…when the opportunity came up again to do the [role] at Waminda I come back … ‘cos it was really Waminda I wanted to work for…”* (HWW10).

### Teamwork and collaboration

Staff felt that they were part of a team of strong, motivated and passionate women who were very solution focused to provide the best services and care to clients and members of the community. Teams were inclusive of each other and worked together to maintain trusting and respectful relationships, supporting one another with the pressures of their roles: *“I love that I’m supported by my team umm, and that we work really well together when it comes to complex issues.”* (HWW03).

Staff reported feeling accepted, and described working for Waminda as *“more like a family than a job [where] everyone’s looking out for everyone”* (HWW08). This reflects the strong family values that are an important part of Aboriginal culture, working together as a family unit to resolve issues and support one another.

Staff valued their ability to work across Waminda programs and share their strengths and passions throughout the service. Staff reported a sense of ownership and pride in the way teams worked together to enable Waminda to keep running despite significant challenges, position vacancies and funding cuts in the past.

Teamwork, building relationships between staff and *“work[ing] collaboratively with other community services…are really important”. “We know that through relationships comes change and, and so there’s a lot of encouragement to work on those relationships”* (HWW05) in order for the organisation to further progress.

### Cultural safety

Staff spoke of the culturally safe working environment at Waminda. They described the significance of Waminda’s foundation based on Aboriginal culture, their focus on spirituality, healing, connection to country and roles as an Aboriginal woman. They noted the ability to feel accepted and respected as a staff member and as a community member.

*“… I think, if other services are looking to improve their retention rates I think they need to probably just identify their workers and them as a whole person, not just a worker in the organisation and coming in and doing this, this, this and this. I mean, especially Aboriginal organisations, they need to look at that person, working, living in communities as well. So, we come with just as much community issues as, as a client.”* (HWW04).

Respondents highlighted that they liked, *“the fact that they’ve got a Cultural Committee … I’ve never heard of another organisation having a Cultural Committee”* (HWW10). Staff also valued Waminda providing either leave for cultural days, or that these were celebrated at the workplace.

*“We have a lot of days of significance that’s recognised by this organisation … a lot of the staff here are Aboriginal women, and I’m an Aboriginal woman, and to have an organisation that recognises your culture is pretty significant. It shows respect… not many organisations do that so, to respect your culture is pretty awesome.”* (HWW03).

Staff felt Waminda’s culture resulted in a work environment, where one expected to be treated as an individual rather than just solely an employee, with the ability to address any issues that arose in a mature and respectful way.

*“… we work really hard and deliberately, on our culture here, so it’s not something that we just hope that we’re gonna get right and we do. We do talk about lateral violence… We try and ensure that people are supportive, not tearing people down, and I think that’s, that’s really, really important, because people come from other organisations or situations where that’s just the norm and everyone just works like that or lives like that, they just sort of stick their head down and hope not to get in the, the bullying or the harassment or whatever it is. So I think that’s why, why here works because you don’t expect to be treated disrespectfully, like you expect to be treated with respect and be included.”* (HWW02).

Staff liked how Waminda recognised the significance of cultural safety for building trust between clients and staff. This was important to staff because of the negative experiences the community had with mainstream health services in the past.

*“…women will probably come here more so than any of the other services around town. The premise, or why the service was initially set up back in 1984, was based on the fact that Aboriginal women experienced such trauma through racism and discrimination that they’d often leave things to the end point and to really dire situations before accessing a service…”* (HWW06).

### Supervision

Supervision supported staff in a number of different forms: role supervision from managers; formal operational supervision with managers; and clinical supervision with a psychologist or counsellor. This comprehensive access to supervision was valued by staff, and allowed them to feel supported from their everyday tasks through to crisis support when emergency supervision was needed. Staff valued the *“even playing field, …you can go and talk to [CEO] if you need to, you can go and talk to someone else…”* (HWW10). Lateral violence existed but was *“nipped in the bud”* (HWW10) before it could become a serious issue.

Staff reported receiving strong support from the CEO. The CEO is a non-Aboriginal person; however, staff expressed her shared deep respect and understanding for Aboriginal culture and the sense of community. Staff felt that her belief in the organisation and employees was evident, and that they were trusted to make their own decisions. They felt she had the best interests of the organisation in mind, was very solution focused, and could help to resolve very difficult and complex issues.

*“I actually love working at Waminda because if there is a problem I can, I have, all staff have the flexibility of getting in contact with our CEO. She’s very approachable and she’s very solution focused and I feel that, that’s needed in an organisation like this, because we deal with a lot of issues every day and sometimes we need that high level of expertise to deal with complex issues. So just having that support from a high level is, is really great.”* (HWW03).

Some staff highlighted their frustration with how ACCHS’ are funded. External pressures due to uncertain funding did affect staff in management positions, who described their dismay at *“having to get money again, having to do all that work …to meet government requirements that don’t make a lot of sense … we still got to jump through a lot of hoops all the time”* (HWW02).

### Professional advancement

Staff valued the professional support Waminda provided to facilitate role promotion and career progression. They noted regular training and study opportunities, the capacity to work on studies during work hours, and the opportunity to attend workshops and conferences. Staff felt valued for being employed for their personal strengths and skills. Additionally, Aboriginal staff felt recognised for their cultural capital and knowledge.

*“Waminda is always supporting us to improve our qualifications and our training and our upskill[ing]. …I come from [non-related employment field] and now I’m a manager. Yeah so, that’s a really big thing for me, and I’ve got five children at home too, so sometimes I think about do I have the capacity to do this, do I have the capacity to do that? Like I, it wasn’t easy doing those [courses], running a household and all that sort of stuff. Like the support that Waminda gave me, giving me study time during work hours and that sort of stuff, yeah the support’s there for us to go in directions that we probably thought that we couldn’t.”* (HWW04).

Some respondents mentioned they were employed at Waminda after completing a practical component of their study at the organisation. While this created employment pathways, some staff mentioned how *“…we really need to make sure that we are getting Aboriginal students where we can”* (HWW10), to ensure *“Aboriginal people come through to run the service”* (HWW04) in the future.

### Recognition

Participants felt recognised for their efforts and work they performed at Waminda. They noted that they felt trusted and supported to make decisions that were best for the organisation, they were given opportunities at staff workshops and planning days to have their ideas heard and considered when decisions were made. These conditions made participants feel supported and treated as a whole person, rather than solely an employee.

*“…we’d have staff meetings and every different process was talked about with all of the staff and their opinions were asked for. So it felt like the staff were really valued and that their opinions and …it felt like you’re being heard.”* (HWW07).

Staff appreciated that they were encouraged to take time for self-care, to ensure they looked after themselves to deliver the best work they could. One respondent mentioned she *“came very close to burning out, about four, nearly five years ago”*. However, with fellow staff voicing their concerns, management were able to put *“things in place…around my role, like what could they do to help my role out…”* (HWW08). Staff had access to paid and unpaid leave, regular exercise and wellbeing programs, and felt they were supported to focus on and maintain physical, emotional and spiritual health at their workplace.

*“…I think that’s the beauty of this place as well is that we know that self-care is the most important thing to be able to improve Community and if you as a, as a worker and as a person are not right then you’re able to take that time and deal with that.”* (HWW04).

Staff reported appreciating how managers *“…check in on us even after hours if she knows we’ve had a stressful day, she’ll send a message or give us a call, she sends us emails saying how great we’re doing…”* (HHW09). They also valued management organising team bonding and self-care activities such as ‘pamper days’ where staff *“just went out we had fun and … know we will all go back tomorrow and everyone will just jump straight back into work”* (HWW08).

Although funding cuts resulted in staff having their hours cut or losing their employment altogether, the CEO navigated these issues skilfully, ensuring trusting and respectful relationships were maintained as well as staff dignity. Letting staff know they were valued, avoided bitterness with staff stating, *“if I could have stayed I would have”* (HWW06).

Waminda acknowledged contribution staff had made by organising afternoon teas and gifts when staff left or their position was affected due to funding, and the sensitive manner in which staff were let go, resulted in some staff returning to the organisation when they were offered their positions back after more funding was received.

*“I’d been laid off I think about three months and I was still at TAFE and I got a phone call on my birthday from [name], … and she was so excited she said “I just want to let you know we can give you your hours back” … she started crying over the phone. I said this is amazing…”* (HWW08).

## Discussion

This case study highlights factors highlighted as being important to retain staff retention factors at one particular Aboriginal Health Service. We add to the literature by identifying the importance of bi-directional communication, as well as demonstrating that social accountability, teamwork and collaboration, cultural safety, supervision, professional advancement, and recognition continue to be important factors that contribute to health and wellbeing staff retention in Aboriginal Health Services.

Many Aboriginal health and wellbeing staff, including those at Waminda, are members of the communities in which they work and feel a sense of responsibility and deep desire to give back to their community [26, 27]. Staff valued working for an organisation whose social accountability and cultural values aligned with their own, was concerned for the greater good of the community, and was accountable for its actions. Having meaningful work is a protective factor against staff burnout [12].

The need for effective teamwork and collaboration in the workplace is well identified [27–29]. Waminda encouraged strong teamwork and collaboration with other services to ensure that community needs were met. Staff felt supported, accepted, and recognised for their work, which increased their retention. These strong relationships create an environment, where staff want to do their best for the benefits of Waminda and the community.

Previous research has identified strong channels of communication as being important in ACCHS’ [27, 30], especially rural and remote settings [31]. Open channels of communication at Waminda meant that staff felt they were being heard and could see their suggestions and feedback being translated back into services for the community. This bi-directional flow of communication between staff and decision makers was a key finding in this study, and has not previously been reported in the literature.

Waminda recognised the benefits of having healthy staff for organisational productivity and individual health [32] and provided a range of additional supports to maintain staff health and wellbeing. Flexible working arrangements and access to wellbeing programs to help support a work-life balance was offered, and access to support staff or counsellors allowed staff to work through issues as needed instead of having to leave their personal issues ‘at the door’. Recognition of staff needs, goals and providing additional supports ensured staff could look after themselves so they could look after the community.

Opportunities for advancing professional skills and qualifications reduces risk of staff turnover [27, 30] by opening up career goals and increasing job satisfaction [11, 27]. Waminda supported and encouraged staff to advance their training and qualifications not only for their personal growth, for improved skill set and abilities within the organisation, but for the benefits they could share with the community as well. While Waminda welcomes student placements, room for improvement to ensuring Aboriginal student placements to build community capacity and create future employment pathways [30] was identified.

Having strong leadership has been identified as important for a stable workforce and retaining staff [27, 28, 30]. This research provides an example of strong leadership guiding the organisation to keep staff and community needs at the forefront. Strong leadership from the CEO kept the organisation progressing and helped to confidently resolve complex issues that were faced regularly in the workplace.

Staff at Aboriginal Health Services are more likely to remain employed if they feel supported and trusted by the Aboriginal community [14, 27, 33]. Staff at Waminda valued working in an atmosphere which resembled a family unit, that promoted mutual respect and trust, important in Aboriginal culture. These cultural values are just as significant for staff as they are for clients [33], who access the service because of its embedded Aboriginal culture which may not be present in mainstream services. Having Aboriginal culture guide Waminda’s operations, was a key enabler to the retention of staff.

### Limitations

This research has some limitations. Participants were female and the majority aged 35–44 years; therefore, the data and described strategies may be less applicable to male health and wellbeing or older staff. Needs also differ between communities and these findings cannot be generalised to all ACCHS’s. However, taking a strengths-based approach may be beneficial in other ACCHS’s and other organisations that have high stress job roles also impacted by high rates of burnout, for example, drug and alcohol workers, and child protection services.

Due to the voluntary nature of this study, staff who were less satisfied with their employment may have been unwilling to participate and their views may not have been captured. Author SD was an employee of Waminda and this could have influenced participant responses. However, rapport and prolonged engagement are strategies to improve rigour in qualitative research and are seen as strengths of this research.

## Conclusions

This study highlighted the factors supporting staff retention at Waminda. We add to the literature by identifying the importance of bi-directional communication, and demonstrate that social accountability, teamwork and collaboration, cultural safety, supervision, professional advancement, and recognition continue to be important factors contributing to staff retention in Aboriginal Health Services. Waminda is a workplace, where staff and organisational values are shared, staff are listened to and suggestions are acted on, and additional support is provided to deal with stress. Waminda provides an environment that fosters the feeling of a family unit by building trusting and respectful relationships, by being accountable to the community, understanding the overlap of being a staff and community member, and with strong leadership. Waminda’s successes provide an important contribution to the body of literature regarding staff burnout and retention within the Aboriginal Health Services. This article identifies unique factors and successful strategies which can help to inform other Aboriginal Health Services with high stress or burnout rates, taking into consideration the individual needs of each service. These findings may be useful for mainstream health services seeking to retain and expand their Aboriginal health workforce. The evidence can inform policy makers and advocate for positive changes to workplace regulations needed for the future. Further strengths-based research is needed to provide broader insight and improvements for staff retention in Aboriginal Health Services.

## Data Availability

The raw data are not publicly available due to the confidentiality of participants.

## Abbreviations

ACCHS: Aboriginal Community Controlled Health Service
CEO: Chief Executive Officer
